# Respiratory Infectious Diseases and Adherence to Nonpharmacological Interventions for Overcoming COVID-19 Pandemic: A Self-Reported Study

**DOI:** 10.1155/2022/4495806

**Published:** 2022-04-07

**Authors:** Sawsan Abuhammad, Omar F Khabour, Karem H Alzoubi, Farah El-zubi, Shaher H Hamaieh

**Affiliations:** ^1^Department of Maternal and Child Health, Faculty of Nursing, Jordan University of Science and Technology, Irbid 22110, Jordan; ^2^Dept. of Medical Laboratory Sciences, Jordan University of Science and Technology, Irbid 22110, Jordan; ^3^Department of Pharmacy Practice and Pharmacotherapeutics, University of Sharjah, Sharjah, UAE; ^4^Department of Clinical Pharmacy, Jordan University of Science and Technology, Irbid, Jordan; ^5^Faculty of Medicine, Jordan University of Science and Technology, Irbid, Jordan; ^6^Deperatment of Community and Mental Health Nursing, Faculty of Nursing, The Hashemite University, Zarqa 13133, Jordan

## Abstract

**Background:**

The aim of the study was to examine changes in the frequency of respiratory diseases during the COVID-19 pandemic and to correlate the changes with nonpharmacological interventions for overcoming the pandemic. In addition, the study explored the predictors of adherence to nonpharmacological interventions among the Jordanian public.

**Method:**

The study is survey-based and self-reported, using convenient sampling. The study was conducted during October–November of 2021.

**Results:**

The study included 1714 participants. About one-quarter of participants reported decreases in the incidence of cold (21.9%), influenza (24.7%), respiratory infections other than cold and influenza (23.3%), tonsillitis (23.0%), and oral ulcers (23.5%). On the other hand, the majority reported no change in the incidence of the above infections (62.0–64.4%). Adherence of the sample to nonpharmacological interventions of COVID-19 was moderate. The percentages of people who always wear a mask, follow social distancing, and use sanitizing procedures were 47.1%, 37.8%, and 68.8% respectively. ANOVA test showed a significant correlation between the incidence of respiratory/oral infections and adherence to nonpharmacological interventions. The multiple regression test showed that people who followed COVID-19 news, have children, have a job, and being married were more adhered to nonpharmacological measures compared to others.

**Conclusion:**

Implementation of nonpharmacological interventions used to overcome the COVID-19 pandemic can be applied to reduce other respiratory infections during their peak seasons.

## 1. Introduction

Since the COVID-19 pandemic began, governments have taken various nonpharmacological measures to prevent disease transmission and reduce mortality [1–3]. Among such efforts include mandatory face masks when in public, frequent hand-sanitizing, and handwashing, social distances, avoiding public gatherings, remote working, and public event cancellation [4, 5]. In addition, reducing contact between individuals by reducing the duration and the number of times they come into contact hence reducing the basic reproducing number and the average number of individuals to whom an infected person can transmit the disease during the incubation period [6, 7]. First countries that experienced the pandemic such as the Philippines, Spain, India, Italy, and Spain have implemented a control measure by locking down cities for months [1, 8, 9]. While other countries such as Turkey, Jordan, and Saudi Arabia had lockdowns running for four days to weeks [10–12].

Hypothetically, COVID-19 measures may also be effective in curbing the spread of other respiratory infectious diseases such as scarlet fever, seasonal influenza, severe acute respiratory illness, and outpatient pneumonia [13–16]. For instance, some respiratory infectious diseases such as seasonal influenza have a huge economic and health impact in the United States [17]. In specific, it causes high morbidity and mortality and increases direct and indirect medical costs because of loss of life [18, 19]. Various studies have shown a significant decline in respiratory disease such as influenza transmission after implementing nonpharmacological interventions in mainland China and Hong Kong [20, 21]. For example, the incidence rate of respiratory diseases such as influenza and flu in China during the 2019–2020 seasons was significantly reduced by 64% compared to past seasons [20]. There was a similar decrease in influenza-related illnesses in the US during spring 2020 compared to past seasons [17, 18]. Although current research has proved the reduction of the spread of respiratory disease due to COVID-19 control measures [22], it is challenging to determine an accurate quantification of the reduced influenza incidence rate in Jordan. Since respiratory disease activities exhibit substantial variations during peak timings and intensity across different seasons, estimating incidence rates of influence is unreliable. Besides, different types of respiratory diseases circulate simultaneously in a specific population with variable seasonal phasing [17]. Also, variations in regional respiratory diseases dynamics and nonpharmacological intervention implementation may have significant geographical disparities in incidence reduction, something that national-level analysis cannot determine [18, 22]. The current investigation aimed to study changes in the frequency of respiratory diseases during the COVID-19 pandemic and to correlate the changes with nonpharmacological interventions for overcoming the pandemic. In addition, the study investigated the predictors of adherence to nonpharmacological interventions among the Jordanian people.

## 2. Method

### 2.1. Study Design

The study adopted a cross-sectional design to achieve the study objectives. Subjects were adults (age: ≥ 18 years old) living in Jordan during the pandemic. The study was conducted during Oct-Nov of 2021 and participants were invited to participate using social media platforms.

### 2.2. Sample and Participants

Adults (over the age of 18) who live in Jordan and speak Arabic fluently met the inclusion criteria. The quota sampling strategy used was based on the participant's age and gender to ensure that the data collected was representative of Jordan's general population. G-Power 3.1., Universitat Kiel, Germany, was used to calculate the sample size for the study, which was based on the convenience/quota sample method, a small effect size, an alpha of 0.05, and a power of 0.95. The minimum number of subjects required was 1300. Out of the 2000 people who began the survey, 1714 completed the study. As a result, the final working number of research subjects was 1714.

### 2.3. Ethical Approval

The study was approved by the Institutional Review Board of Hashemite University. Participants consented online before they started filling the study instrument. The participants were assured that the questionnaire is anonymous, and the data will be used for research purposes, and they have the right to skip any question that they do not want to answer.

### 2.4. Study Instruments

The study instrument was developed by the research team and was face validated by experts in the field. The instrument was pilot-tested on 30 participants to ensure its clarity and consistency. After revising the instrument based on the pilot study, the questionnaire was uploaded to Google forms. The questionnaire was divided into different parts. The first part was an introductory part that included information about the study, eligibility to participate, and an online informed consent. The second part asked questions related to the demographics of the participants such as age, education, sex, work status, health insurance, and others. The third part asked questions related to changes in the frequency of respiratory infections during the COVID-19 pandemic. These include cold, influenza, tonsillitis, oral ulcer, and acute respiratory illnesses. For the participants to differentiate between the types of respiratory problems. The authors provided in detail for each respiratory problem within the survey. The respondents were given the choices “increase”, “no change”, and “decrease” when answering the questions to the third part. The last part asked questions related to the magnitude of adherence of the participants to nonpharmacological interventions against COVID-19 adopted in Jordan. The link of the questionnaire along with information about the study was distributed online through social media platforms. After the participants had responded to the questions. The researchers then check the data for consistency and completeness. Data cleaning was performed, and data were entered in computer using Excel datasheets.

### 2.5. Data Analysis

The SPSS software version 25 was used to analyze the data. Descriptive statistics was used to describe demographical variables such as age, gender, education, income, and social status. ANOVA test was used to predict the difference in the incidence of many respiratory infectious diseases based on the degree of adherence to nonpharmacological measures. Multiple regression was used to find factors that impact degree of adherence to nonpharmacological measures against COVID-19.

## 3. Results

### 3.1. Demographical Characteristics of the Participants

The demographic of participants is shown in Table 1. The mean age of participants was 31.7 (SD = 9.8). The number of females was 1158 (67.4) and the number of was males 556 (32.4). The majority have health insurance (75.0%), have middle income (61.4%), married (52.5%), and live in a city (64.2%).

### 3.2. Description of Changes in the Frequency of Respiratory Diseases during the COVID-19 Pandemic

About a quarter of participants reported a decrease in respiratory and oral infections during the pandemic (Table 2). This includes cold (21.9%), influenza (24.7%), acute respiratory infections (23.3%), tonsillitis (23.0%), and oral ulcers (23.5%).

### 3.3. Adherence to Nonpharmacological Interventions during COVID-19 Pandemic

Table 3 shows the adherence of the participants to nonpharmacological interventions during the COVID-19 pandemic. The findings showed that adherence ranged from moderate to high with mean adherence scores between 3.54 and 4.3 (Table 3). The number of participants who always wear a mask, follow social distancing, and use sanitizing/hand washing were 47.1%, 37.8%, and 68.8% respectively. The percentage of people who do not adhere to nonpharmacological interventions were less than 10%.

### 3.4. Correlation between Adherence and Respiratory Diseases

ANOVA test was conducted to determine the correlation between changes in the frequency of infectious respiratory/oral diseases and the mean score of adherence to nonpharmacological measures. All listed diseases in Table 4 were correlated significantly with adherence to nonpharmacological measures. The highest was for tonsilitis (*F* = 3.64, *P*=0.004) and influenza (*F* = 3.023, *P*=0.004).

### 3.5. Multivariate Regression Analysis of the Predictors of Adherence to Nonpharmacological Interventions during the COVID-19 Era

The regression model (Table 5) was significant (*F* = 6.78, *P*=0.04). This means that many factors predicted adherence to nonpharmacological measures and these factors were following COVID-19 news (*B* = 0.099, *P* ≤ 0.001), having children (*B* = 0.092, *P* < 0.039), working status (*B* = .078, *P*=0.008), and social status (*B* = 0.123, *P* < 0.04). This means that people who hear news frequently, have children, have a job, and are married adhere to nonpharmacological measure compared to others.

## 4. Discussion

The first incidence of COVID-19 was in China in December 2019. The virus quickly spread across the world, attracting global attention as an international public health crisis. Governments implemented various control measures such as compulsory masks in public, hand washing/sanitizing, social distancing, border controls, and the close of schools. These control measures, or what is called nonpharmacological measures, were implemented to reduce the transmission of the virus. In theory, other transmissible respiratory infections can be also reduced in the communities by the implementation of such measures. The current study using a self-reporting survey investigated changes in the frequency of respiratory infections and the degree of adherence to nonpharmacological measures.

The results showed that about a quarter of participants reported a decrease in the frequency of respiratory/oral infections that include cold, influenza, oral ulcer, tonsillitis, and acute respiratory infections during the pandemic compared to previous years. This finding agrees with previous studies that reported a decrease in airborne infections such as pneumonia, enterovirus, mycobacterium, adenovirus, and tuberculosis during the pandemic. For example, a study conducted in Korea revealed that influenza transmission was declined by 27% to 39% during COVID-19 outbreaks. In another study from Taiwan, zero cases of measles were reported during the COVID-19 pandemic, which was attributed to pharmacological measures [23]. In Pakistan, a 50% reduction in measles was also reported during the pandemic compared to previous periods [24]. A survey conducted in Taiwan also revealed a significant drop in tuberculosis cases during the COVID-19 pandemic [25]. Thus, droplet aerosol precaution and preventive measures that have participated in containing COVID-19 transmission might lead to similar benefits by controlling other respiratory infectious diseases [18, 22].

Among the control measures for the prevention of COVID-19 spread were social distancing, using masks in public, and frequent hand-sanitizing/washing [15, 21]. In the current study, the adherence of the public to control measures was moderate to high. The percentages of people who always wear a mask, implement social distance, and wash hands/sanitizing all the time were 47.1, 37.8%, and 68.8%, respectively. The people who do not adhere at all to precautions were less than 10%. In a study that was conducted in North America and Europe, adherence to social distancing was between 40% and 90% [26]. In a USA study, adherence to the Center for Disease Control and Prevention guideline was generally high with suboptimal adherence to social distancing and hygiene practices in younger adults [27]. Similar findings were reported in studies conducted in the United Kingdom, Kuwait, and China [2, 28, 29]. Among health care providers, a study found that using masks was 100%, and practicing hand washing/sanitizing was optimal [30]. However, the public adherence to masks and hand washing is expected to be not optimum as in the case of healthcare workers.

In our study, correlations between changes in the frequency of respiratory infectious diseases and adherence to nonpharmacological measures were reported. These include cold, influenza, and tonsillitis. Various studies have shown a significant decline in respiratory diseases such as influenza transmission after the implementation of nonpharmacological interventions in mainland China and Hong Kong [20, 30]. For example, the incidence rate of respiratory diseases such as influenza in China during the 2019–2020 seasons was significantly reduced by 64% compared to past seasons [20]. There was a similar decrease in influenza-related illnesses in the US during spring 2020 compared to past seasons [17, 18].

The current study reported several factors that predict adherence to nonpharmacological measures during the COVID-19 pandemic in Jordan. Among identified factors are following COVID-19 news, having children, working status, social status, and income. However, gender was not among the predictor factors. The age showed marginal significance with a *P* value of 0.053. In a previous study conducted in Croatia, women of older age were found more likely to adhere to the non-pharmacological measures of COVID-19 [31]. In a study that was conducted in Somalia, female gender, type of work, being a student, obtaining COVID-19 information from official sources, and higher education were strong predictors to nonpharmacological measures [32]. In a study from Uganda, living in a city, age, and receiving COVID-19 information from health workers were among predictors to nonpharmacological measures [33]. In a Canadian study, age, female gender, social status, having a job, and having children were more adherent to social distancing than others [34]. Thus, the predictors of adherence to different measures taken by the governments in the different populations might depend on the culture and the nature of the population.

The present findings could be used by healthcare policymakers in Jordan to reduce the burden of infectious respiratory diseases in Jordan. Identification of predictors of adherence to nonpharmacological interventions provide feedback to health care authorities to implement better preventive interventions.

### 4.1. Limitations

This study does have some limitations. For instance, this study employed a cross-sectional design, which limits the ability to provide a broad reference for a cause-and-effect relationship. Second, the data were self-reported, which reduces the study's reliability. Another limitation is the low response in comparison to the methods. However, it is worth noting that a low response rate is inherent in online self-administered surveys, where participants feel less obligated to complete the survey than in-person data collection methods.

## 5. Conclusion

In conclusion, the study reported the change in the frequency of respiratory/oral infections during the COVID-19 pandemic. Adherence to nonpharmacological interventions of COVID-19 can be useful to reduce other respiratory infections during their peak seasons.

## Figures and Tables

**Table 1:** Demographical characteristics of the participants (*N* = 1714).

Items		Category	Frequency	Percent
**Gender**		Male	556	32.4
		Female	1158	67.6
**Insurance**		No	428	25.0
		Yes	1286	75.0
**Working status**		I Do not work	486	28.4
		Full time	672	39.2
		Part time	166	9.7
		Student	390	22.8
**Income (JD) (1$USA** **=** **0.71 JD)**		≤400	550	32.1
		401–800	1053	61.4
		>800	111	6.5
Educational level	School certificate		276	16.1
	Diploma		182	10.6
	University student		284	16.6
	Bachelor's		734	42.8
	Master/PhD		238	13.9
**Social status**	Single		772	45.0
	Married		899	52.5
	Divorced		43	2.5
**Do you have a child**	No		863	50.4
	Yes		835	48.7
**Living areas**	City		1100	64.2
	Village		614	35.8
**Smoking**	No		1058	61.7
	Yes		656	38.3

**Table 2:** Description of incidence of some respiratory diseases (*N* = 1714).

Respiratory disease	Decreased: *N*(%)	No change: *N*(%)	Increased: *N*(%)
Influenza	260 (24.7)	671 (63.8)	121 (11.5)
Cold	229 (21.9)	662 (63.3)	155 (14.8)
Acute respiratory infections	243 (23.3)	647 (62.0)	154 (14.8)
Tonsillitis	241 (23.0)	656 (62.7)	150 (14.3)
Oral ulcers	244 (23.5)	669 (64.4)	126 (12.1)

**Table 3:** Adherence to nonpharmacological interventions during COVID-19 pandemic.

Item	Seldom adhere	Sometimes adhere	Adhere always	Mean score^*∗*^
Wearing mask: *N*(%)	102 (7.2)	644 (45.6)	665 (47.1)	3.80
Social distancing: *N*(%)	149 (10.6)	723 (51.6)	530 (37.8)	3.54
Washing hand: *N*(%)	51 (3.6)	388 (27.6)	968 (68.8)	4.30

^*∗*^Mean score was calculated by giving 1 point to seldom adhere, 3 points to sometimes adhere, and 5 points to always adhere.

**Table 4:** Correlation between several respiratory infectious diseases and nonpharmacological interventions.

Item		Sum of squares	Mean square	F	Sig.
**Influenza**	Between groups	7.560	1.080	3.023	0.004
	Within groups	603.348	0.357		
	Total	610.907			
**Cold**	Between groups	7.821	1.117	3.004	0.004
	Within groups	625.278	0.372		
	Total	633.099			
**Acute respiratory infection**	Between groups	5.376	0.768	2.017	0.050
	Within groups	637.430	0.381		
	Total	642.806			
**Tonsillitis**	Between groups	9.279	1.326	3.649	0.001
	Within groups	610.314	0.363		
	Total	619.592			
**Oral ulcers**	Between groups	5.598	0.800	2.261	0.027
	Within groups	589.961	0.354		
	Total	595.558			

**Table 5:** Multivariate logistic analysis of the predictors of adherence to nonpharmacological interventions during COVID-19 pandemic.

Model	B	Std.	Beta	*t*	Sig.
(Constant)	6.993	0.554		12.627	≤0.001
Age	0.010	0.005	0.065	1.934	0.053
Gender	−0.004	0.089	−0.001	−0.042	0.967
Insurance	−0.053	0.093	−0.015	−0.571	0.568
Working status	−0.082	0.031	−0.078	−2.657	0.008
Income	0.026	0.044	0.183	2.887	0.004
Educational level	−0.003	0.030	−0.003	−0.100	0.920
Social status	0.350	0.121	0.123	2.887	0.004
Do you have a child	−0.284	0.137	−0.092	−2.070	0.039
Living areas	−0.039	0.080	−0.012	−0.488	0.626
Smoking	−0.129	0.084	−0.041	−1.545	0.122
Following COVID-19 news	0.623	0.167	0.099	3.733	≤0.001

## Data Availability

The data used to support the findings of this study will be available from the corresponding author upon request.
